# Perception of pharmacy students toward numeracy: An observational study from King Saud University, Riyadh Saudi Arabia

**DOI:** 10.3389/fpubh.2022.1014328

**Published:** 2022-11-14

**Authors:** Ziyad Alrabiah, Azher Arafah, Muneeb U. Rehman, Wajid Syed, Salmeen Babelghaith, Abdulrahman Alwhaibi, Sultan Alghadeer, Abdulaziz Alhossan, Mohamed N. Al Arifi

**Affiliations:** Department of Clinical Pharmacy, College of Pharmacy, King Saud University, Riyadh, Saudi Arabia

**Keywords:** numeracy, pharmacy students, Saudi Arabia, perceptions, health care

## Abstract

**Background and objective:**

Numeracy is the branch of mathematics involved in understanding basic calculations, quantitation, estimation, reasoning, and execution of multistep operations. It is very imperative that pharmacists understand and apply numeracy skills in their routine work in the interest of their profession and patient care. This observational study was designed to assess the pharmacy student's perceptions of numeracy.

**Methods:**

A prospective observational study was conducted by the Department of Pharmacy, King Saud University, Kingdom of Saudi Arabia, between December 2021 and February 2022. All the enrolled subjects pursued a 5-year Pharma degree course at the university using a 9-item instrument, which accessed the perception of students toward numeracy. The data were analyzed using the statistical software statistical package for social science (SPSS) version 26.0 (SPSS Inc., Chicago, IL, USA). Chi-square and Fisher's exact test were used to derive an association between various parameters of the study subjects. A *P*-value of < 0.05 was taken as statistically significant.

**Results:**

A total of 550 pharmacy students were approached in this study, out of which 21 (3.8%) students were excluded due to incompleteness of the responses; thereupon, 529 students were included in the study. We learned that almost 90.0% of students had excellent and/or good mathematical ability, but at the same time, they were frequent users of calculators. Most of the students endorsed the importance of numeracy and showed their interest in attaining more knowledge of numeracy. Similarly rating the perceptions of mathematical ability is significantly associated with the frequency of use of a calculator for calculations (*p* = 0.0001).

**Conclusion:**

Pharmacy students showed interest in numeracy and correspondingly showed excellent perceptions toward mathematical ability. Although the role of numeracy has been well accepted, inciting changes in teaching-learning practices through mathematically focused teaching approaches throughout the pharmacy program will increase its applicability in healthcare.

## Introduction

Mathematics plays an interdisciplinary role in medical, biological, chemical, physical, and earth sciences (1), and its application extends to pharmacy courses. Due to its importance, mathematics is a crucial subject in the pharmacy curriculum. Mathematics provides knowledge and skills vital in altering technological scenarios (2). Numeracy is proficiency in mathematics or the ability to use or apply mathematics. Numeracy includes all those skills needed to decipher appropriate mathematical models for solving fundamental and intricate problems, especially in medical sciences (3). Inadequate numeracy knowledge has been associated with worse health indicators and consequences, including increased hospitalizations, inferior disease diagnostic facilities, more significant occurrence and severity of chronic diseases, non-adherence to higher medication, and lesser health behavior engagement (4, 5). Limited numeracy skills are also associated with lesser knowledge of disease risk factors (6, 7).

The quality of numeracy of pharmacy students was first questioned in 2000, after “The Peppermint Water case,” which led to the death of a 3-week-old boy in 1998, due to the addition of the excess amount of chloroform, by a pharmacist, to peppermint water prescribed to treat colic of a 4 days old baby. This incident raised queries about the knowledge and readiness of pharmacy undergraduates regarding the calculation of quantities of constituents in medication (8). Subsequently, it has been described that pharmacy students have lower numeracy skills at university levels and almost rely on calculators leading to skewed calculations and an inability to solve numerical problems encountered during and after the completion of the course (9). Keeping in view the importance of numeracy, the Royal Pharmaceutical Society of Great Britain (RPSGB), The Pharmaceutical Society of Northern Ireland (PSNI), and The School of Pharmacy at Queen's University Belfast (QUB) have introduced an obligatory section containing numerical questions in the entrance exam paper of pharmacy degree course way back in 2002, 2005, and 2010, respectively (10).

As per previous studies, competence in mathematics plays a significant role in forecasting the success of pharmacy institutes (11). Basic math skills have been linked previously to academic enactment among pharmacy students (12, 13). Many studies reported that pharmacists could employ their numeracy skills as part of their professional practice in dispensing and compounding dosage forms, ultimately leading to better patient care (14, 15). Studies have shown a higher frequency of calculation deficiencies and relatively decreased mathematical ability in medical students, undergraduate pharmacy students, and doctors (16–18). Therefore, a good grasp of numeracy is fundamental to allow healthcare professionals, especially pharmacists, to use this knowledge for executing mathematical functions such as drug-dose calculations (19).

Thus, this study aimed to evaluate the numeracy skills of pharmacy students seeking their undergraduate degree course at King Saud University, Saudi Arabia. Additionally, students' level of confidence and attitude toward mathematics was assessed, which will ultimately perceive their mathematical performance. This study would identify simple indicators to better understand how pharmacy institutions and educators can appraise themselves to achieve academic excellence. Therefore, this study aimed to assess pharmacy students' perceptions of numeracy.

## Materials and methods

### Study design

It was a prospective observational study conducted by the Department of Pharmacy, King Saud University, Kingdom of Saudi Arabia, between December 2021 and February 2022. All the enrolled subjects pursued a 5-year Pharma degree course at the university.

### Questionnaire

The questionnaire used for this study was adopted based on previous studies published in a similar context (13, 14). The questionnaire was designed initially in the English language to assess the perceptions of Pharmacy students toward numeracy; then, the questionnaires were translated into the Arabic language with the help of native Arabic speakers using forward and backward translation procedures. The questionnaire was divided into two parts. In the first part, the socio-demographic data such as age, gender, pharmacy year, and nationality were recorded. The second part of the study contained one item asking for the rating of student perceptions toward their mathematical skills (excellent/fair/good/poor). The third part of the study asks participants nine multiple-choice questions to access students' perceptions of numeracy. Before beginning the study, first, a research specialist in the associated field evaluates the first draft of the tool, second, a pilot study was done with a randomly selected sample of 20 students to get their feedback. The final draft of the questionnaires was distributed and translated into the local language with help of native Arabic speakers then the tool was sent to the intended participants, with changes based on the pilot research. The reliability test was performed using SPSS to calculate Cronbach's alpha, and a value of 0.70 suggested questionnaires adequate for the study. The pilot study's data were not used in the final analysis.

## Statistical analysis

The data were analyzed using the statistical software SPSS version 26.0 (SPSS Inc., Chicago, IL, USA). Chi-square and Fisher's exact test were used to derive an association between numeracy and socio-demographic parameters of study subjects. A *P*-value of < 0.05 was taken as statistically significant.

## Results

### Study participants

A total of 550 pharmacy students were approached in this study, out of which 21 (3.8%) students were excluded due to incompleteness of the responses; thereupon, 529 students were included in the study. The response rate of pharmacy students in the study was 96% and included those who thoroughly answered the questionnaires. Out of 529 subjects, 288 (54.4%) were men and 236 (44.6%) were women. The majority of study subjects were aged between 18 and 25 years (96.2%). Fifty-three (10.0%), 84 (15.9), 102 (19.3), 78 (14.7), 68 (12.9), and 144 (27.2) subjects enrolled in the study were studying in the first, second, third, fourth, fifth, and last year of pharmacy course. Most enrolled subjects were Saudi nationals 512 (96.7%). Characteristics of participants are shown in Table 1.

**Table 1:** Characteristics of study participants.

**Variables**	***N*** **= 529 (%)**
**Gender**	
Male	288 (54.4)
Female	241 (45.6)
**Age group**	
>18 years	07 (1.3)
18–25 years	509 (96.2)
26–30 years	13 (2.5)
**Pharmacy year**	
First	53 (10.0)
Second	84 (15.9)
Third	102 (19.3)
Fourth	78 (14.7)
Fifth	68 (12.9)
Final	144 (27.2)
**Nationality**	
Saudi	512 (96.7)
Non Saudi	17 (3.2)

### The attitude of pharmacy students toward numeracy

The response of Pharmacy students to various questions about numeracy is given in Table 2. According to our survey, the majority of the students (461 of 529; 87.1%) agreed that they study “A” grade mathematics during their pharmacy course, while 77.7% (411 of 529) agreed that they learn numeracy during graduation. Regarding the importance of mathematical calculations in the future, most of the students endorsed its importance (428 of 529; 81.0%). In this study, more than half of the students sometimes used a calculator for carrying out calculations (313 of 529; 59.2%), while 37.3% (197 of 529) used it always, and only 3.2% (17 of 529) used calculators rarely. Regarding teaching numeracy skills in college, there was a mixed response among students. When asked about the support provided by the college in solving mathematical problems, 28.9% responded positively and 24.3% responded negatively. Nearly 40.0% of the students revealed that there should be more emphasis placed on pharmaceutical calculations within the Pharma degree course. Additionally, 41.4% of students believed that they would benefit from extra classes in basic mathematics. When asked about the ability to perform mathematical calculations in the pharmacy degree course, 34.8% of them reported an increased ability to perform calculations, while as 48.4% reported no change (Table 2).

**Table 2:** Perceptions of pharmacy students toward numeracy.

**Questions**	***N*** **= 529 (%)**
**Did you study A-level math?**	
Yes	461 (87.1)
No	68 (12.9)
**Did you learn about numeracy? (mathematical calculations in healthcare)**	
Yes	411 (77)
No	111 (20)
**Do you feel that the ability to perform mathematical calculations will be important to you as a future doctor?**	
Yes	428 (81.0)
No	101 (19.0)
**How often do you use a calculator when carrying out calculations?**	
Always	197 (37.3)
Sometimes	313 (59.1)
Rarely	17 (3.2)
Never	02 (0.4)
**Do you feel that there is adequate teaching provided by your college in relation to numeracy skills needed to complete the bachelor's degree?**	
Yes	295 (56.0)
No	234 (44.0)
**Do you think there is sufficient support within your college in relation to problems encountered by a student with calculations?**	
I do not know	248 (46.8)
Yes	153 (28.9)
No	128 (24.3)
**Do you think there should be more of an emphasis placed on pharmaceutical calculations within the Pharma degree course?**	
I do not know	140 (25.4)
Yes	208 (39.4)
No	181 (34.2)
**Do you feel that you would benefit from extra classes in basic mathematics?**	
I do not know	158 (29.8)
Yes	219 (41.4)
No	152 (28.8)
**Since starting your bachelor's degree do you feel more or less able to perform mathematical calculations?**	
Less able	89 (16.8)
More able	184 (34.8)
No change	256 (48.4)

### Association between frequency of calculator use and characteristics of study subjects

Table 3 describes the association between the frequency of use of the calculator and the characteristics of study subjects. The findings reported no significant association between students' characteristics and the frequency of calculator use (*P* > 0.005). Similarly, studying a level of mathematics is not significantly associated with the frequency of calculator use (*p* > 0.005).

**Table 3:** Association between the frequency of use of calculator and characteristics of study subjects.

**Demographics**	***N*** **= 529 (%)**	**Frequency of use of calculator when carrying out calculations**				
		**Always**	**Sometimes**	**Rarely**	**Never**	***P*** **-value**
		**197 (37.3)**	**313 (59.1)**	**17 (3.2)**	**02 (0.4)**	
**Gender**						0.5
Male	288 (54.4)	119 (41.3)	159 (55.2)	09 (3.1)	01 (0.3)	
Female	241 (45.6)	78 (32.2)	154 (64.0)	08 (3.4)	01 (0.4)	
**Age group**						0.424
>18 years	07 (1.3)	02 (28.6)	04 (57.1)	01 (7.7)	00 (0.0)	
18–25 years	509 (96.2)	191 (37.5)	301 (59.1)	15 (2.9)	02 (0.4)	
26–30 years	13 (2.5)	04 (30.8)	08 (61.5)	01 (7.7)	00 (0.0)	
**Pharmacy year**						0.128
First	53 (10.0)	20 (37.7)	31 (58.5)	02 (3.8)	00 (0.0)	
Second	84 (15.9)	27 (32.1)	52 (61.9)	05 (6.0)	00 (0.0)	
Third	102 (19.3)	29 (28.4)	71 (69.6)	02 (2.0)	00 (0.0)	
Fourth	78 (14.7)	28 (35.9)	49 (62.8)	01 (1.3)	00 (0.0)	
Fifth	68 (12.9)	26 (38.2)	38 (55.9)	04 (5.9)	00 (0.0)	
Final	144 (27.2)	67 (46.5)	72 (50)	03 (2.1)	02 (0.4)	
**Did you study A-level maths?**						0.292
Yes	461 (87.1)	168 (42.6)	274 (59.4)	17 (3.7)	02 (0.4)	
No	68 (12.9)	29 (42.6)	39 (12.5)	0 (0.0)	0 (0.0)	

### Rating of student perceptions toward their mathematical skills and characteristics of participants

**Figure 1:**
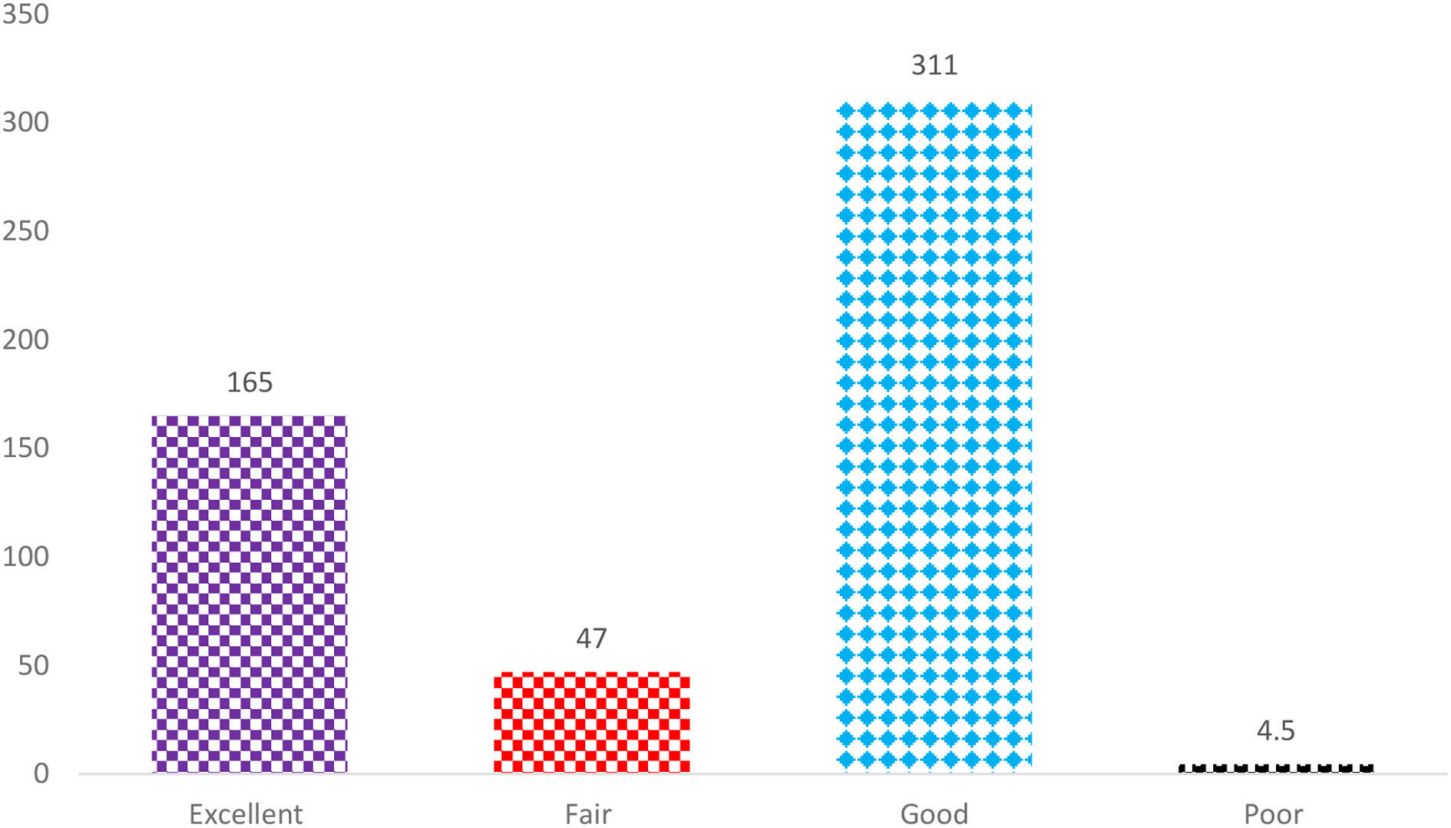
A rating of student perceptions toward their mathematical skills.

Perceptions of students undergoing pharmacy undergraduate degree courses toward their mathematical skills were categorized into Excellent, Fair, Good, and Poor. We observed that almost one-third of study participants believed that they had excellent perceptions toward mathematical ability (31.6%; 167 of 529), more than half had good (58.8%; 311 of 529); 8.8% (47 of 529) had fair, and only 0.8% (04 of 529), respectively (Figure 1). Subsequently, Table 4 shows a rating of student perceptions toward their mathematical skills and characteristics of participants. We did not find any significant association between them (*P* > 0.005). Similarly rating the perceptions of mathematical ability is significantly associated with the frequency of use of a calculator for calculations (*P* = 0.0001) as shown in Table 4.

**Table 4:** Association between the rating of perceptions toward mathematical ability and characteristics of participants.

**Demographics**	***N*** **= 529 (%)**	**Rating of perceptions toward mathematical ability**				
		**Excellent**	**Good**	**Fair**	**Poor**	***P*** **–value**
		**167 (31.6)**	**311 (58.8)**	**47 (8.8)**	**04 (0.8)**	
**Gender**						0.48
Male, *n* (%)	288 (54.4)	102 (35.4)	162 (56.3)	22 (7.6)	02 (0.7)	
Female, *n* (%)	241 (45.6)	65 (26.3)	149 (61.9)	25 (10.6)	02 (0.8)	
Age group						
>18 years, *n* (%)	07 (1.3)	03 (42.9)	04 (57.1)	00 (0.0)	00 (0.0)	0.15
18–25 years, *n* (%)	509 (96.2)	159 (31.3)	303 (59.5)	44 (8.6)	03 (0.6)	
26–30 years, *n* (%)	13 (2.5)	05 (38.5)	04 (30.8)	03 (23.1)	01 (7.7)	
**Pharmacy year**						
First, *n* (%)	53 (10.0)	15 (28.3)	33 (62.3)	05 (9.4)	00 (0.0)	0.594
Second, *n* (%)	84 (15.9)	35 (41.7)	46 (54.8)	03 (3.6)	00 (0.0)	
Third, *n* (%)	102 (19.3)	36 (35.2)	55 (54.0)	10 (9.8)	01 (1.0)	
Fourth, *n* (%)	78 (14.7)	23 (28.4)	44 (56.5)	10 (12.8)	01 (1.3)	
Fifth, *n* (%)	68 (12.9)	20 (29.4)	44 (64.7)	04 (5.9)	00 (0.0)	
Final, *n* (%)	144 (27.2)	38 (25.7)	89 (61.8)	15 (10.4)	02 (1.4)	
**Frequency of use of calculator when carrying out calculations**						0.0001
Always, *n* (%)		43 (26.1)	122 (39.2)	29 (61.7)	02 (50.0)	
Sometimes, *n* (%)		112 (67.9)	183 (58.8)	16 (34.0)	01 (25.0)	
Rarely, *n* (%)		09 (5.5)	06 (1.9)	02 (4.3)	0.0 (0.0)	
Never, *n* (%)		0.0 (0.0)	0 (0.0)	0.0 (0.0)	1.0 (25.0)	

## Discussion

This study was conceived to understand the perception of pharmacy students toward numeracy and to deduce their mathematical ability. Previous studies have examined the extent to which mathematical ability correctly recognizes individuals with insufficient health knowledge compared to a benchmark (20). Others studies have related measures of numeracy (21) or compared measures of health literacy and numeracy (22). Such assessments are planned to help researchers and healthcare providers identify pharmacy students with inadequate health literacy so that timely interventions could be made for the production of a well read breed of pharmacists. There are many occasions, where public pharmacists utilize their mathematical ability as a part of their professional expertise. These comprise dose measurement, and calculating quantities and concentrations. It is, therefore, very much imperative that pharmacists are able to do calculations precisely and habitually so as to safeguard patient safety and the reputation of their profession. The RPSGB Accreditation Document (23) and the QAA subject benchmark statement for pharmacy (24) have made pharmaceutical numeracy mandatory for the attainment of a pharmacy degree.

The age group of study subjects was between 18 and 30 years as the target population was pharmacy students undergoing undergraduate degree courses. In our study, student feedback was recorded in response to the battery of nine questions. Feedback is a serious element of the learning process (25). Nonetheless, making the best use of feedback opportunities would likely increase students' learning opportunities (26).

In our survey, almost 90.0% of pharmacy students had studied A-level maths before entering into pharmacy courses which are in line with the study done in Northern Ireland (14). Our observation is in disparity with a previous study from a pharmacy school that observed a decline in the proportion of entrants with A-level maths over recent years (18). As studies have observed a fall in numeracy standards of university entrants (27) many concerns have been raised about the decline in the number of students taking mathematics as an A-level subject at intermediate levels (28). As most of our pharmacy course entrants were well versed in previous math courses, they were fully prepared for entry into practice in the community. The statement that entry qualifications are a predictor for numeracy proficiency is consolidated by the fact that almost all the pharmacy school entrants had acquired A-level math training in their intermediates and subsequently they performed very well during the period of their degree. Currently, universities complying with regulatory body requirements for pharmacy courses merely need to warrant that the selection criteria of students inculcate basic numeracy skills.

Almost 90.0% of pharmacy students were of the opinion that the ability to perform mathematical calculations will be important as a future doctor. The results demonstrated that students adored mathematics as they realized its scope in medical sciences. Our observations are in coherence with the study by Van der Bergh (29) who found a significant influence of students' interest on the ability to successfully learn and apply mathematics in medical science. The study of Syyeda (30) also backs our outcome that students cherished mathematics. In contrast, a previous study on nurse and midwifery students highlighted that students had a negative attitude toward mathematics and lacked numeracy skills (19) which are further supported by findings of Axe (31) and Wright (32). Various approaches to enhance the interest of pharmacy students in numeracy and to instill confidence have been adopted which include the introduction of numeracy tests in formative assessment and this approach has been found to increase numeracy skills (33).

It was noteworthy that the majority of pharmacy students feel that they would benefit from extra classes in basic mathematics. With the addition of new subjects to the B. Pharma Program, the implementation of extra classes is rather difficult in view of time constraints. However, the university is planning to supplement the students with online supplementary material on applied mathematics thereby improving the numeracy standards. This supplementary material will provide further instruction in basic numeracy and pharmaceutical calculations necessary for better implementation of pharmacy in day-to-day life (14).

In our study, only around 4.0% of students rarely and/or never used calculators (Table 3). Previous data reveals that the use of calculators resulted in an enhanced inclination toward mathematics (34) which provided the impetus for mentors to teach and examine students using this tool. A study by Chamblee et al. reported a significant improvement in the exploration and implementation of technology through the use of graphic calculators (35). Another study reported the beneficial use of calculators to pharmacy students with a basic knowledge of mathematical facts (36). Individual's belief is one of the strongest factors responsible for the low use of calculators (37). Thus, we as teachers, educators, and healthcare providers must have a positive attitude toward the use of gadgets such as calculators to make mathematics meaningful and more applicable to disciplines like medicine and pharmacy in which not only accuracy but the time needed to perform calculations is very much decisive to safeguard the life and interests of a patient.

In our study, the mathematical ability of more than 90.0% of pharmacy students has been rated as either “good” or “excellent” (Table 4). In order to enhance the mathematical ability and to increase the behavioral and cognitive learning of pharmacy students the factors of Walberg's Theory of Productivity should be considered (38, 39). The first group of factors includes aptitude variables that cover students' drive and prior accomplishments. The second group of factors includes those instructional habits that affect students' wisdom of mathematics, especially the amount and superiority of directions. The amount of directions is defined by the extent of time students spend learning mathematics. The superiority of directions includes the degree to which the topic is clear to students, various aspects of presentation, and clarity of language (39). Psychological or environmental factors comprise the last group of factors influencing mathematical ability (39, 40). Lack of mathematical ability among pharmacists is worrisome since it could result in serious errors in dose calculations, considering today's students are tomorrow's professionals (41–44) leading to dire consequences and even the death of the patient (19).

On stratification, we found that first- and second-year pharmacy students and students of younger age groups had higher mathematical ability compared to their seniors which deduce that more attention toward numeracy and pharmaceutical calculations be paid to students in their B. Pharma Program. Our findings may be due to the fact that first- and second-year students receive extensive instruction and classes in numeracy and more than 8 h of teaching time per week is dedicated to numeracy at the beginning of the Program followed by a multiple-choice assessment to check the progress. In addition, first- and second-year pharmacy students are taught mathematics properly for efficient dose measurement and dispensation. During the last years of the pharmacy Program, numeracy calculations performed by students are applicable to day-to-day life rather than the basic calculations used for dose measurements (14). In contrast to our observations, previous studies have reported that first-year midwifery and nursing students had relatively lower mathematical ability (45–47) which hints toward the fact that students entering midwifery and nursing courses lack basic numeracy skills and concepts which may be due to the fact that they did not receive A-level math's training in their intermediate before entering nursing school.

## Limitations of the study

In our study, only self-reporting was noted. In order to do away with this limitation in the future, we would include more than one data source, i.e., data collected through classroom observations; teacher reports; transcripts; and grades of students.

## Conclusion

Competence in numeracy is a requirement for many regulatory accreditation bodies and is necessary for the pharmacy curriculum. According to a self-estimated questionnaire, the pharmacy students had a positive attitude toward numeracy and exceptional or good mathematical ability. Regarding the importance of mathematical calculations in the future, most of the students endorsed its importance additionally more than half of the students sometimes used a calculator for carrying out calculations. Slightly less than half of the students revealed that there should be more emphasis placed on pharmaceutical calculations within the Pharma degree course. Lack of numeracy among pharmacy undergraduate students might increase the risk of potential patient harm once they enter clinical practice. Teachers need to warrant that proficiency in numeracy is reflected while practicing pharmacy and, at the same time, ensure full support to students. This is likely achieved with problem-based teaching practices and a correctly constructed curriculum.

## Data availability statement

The raw data supporting the conclusions of this article will be made available by the authors, without undue reservation.

## Ethics statement

The studies involving human participants were reviewed and approved by IRB-E-21-6348. The patients/participants provided their written informed consent to participate in this study.

## Author contributions

All authors equally contributed to the conception, design, data analysis and interpretation, drafting of the manuscript, and approval of the final version of the article for publication.

## Funding

The authors of this study extend their appreciation to Researchers Supporting Project (Project number RSP-2021/81), King Saud University, Saudi Arabia.

## Conflict of interest

The authors declare that the research was conducted in the absence of any commercial or financial relationships that could be construed as a potential conflict of interest.

## Publisher's note

All claims expressed in this article are solely those of the authors and do not necessarily represent those of their affiliated organizations, or those of the publisher, the editors and the reviewers. Any product that may be evaluated in this article, or claim that may be made by its manufacturer, is not guaranteed or endorsed by the publisher.
